# Isoprene production by *Escherichia coli* through the exogenous mevalonate pathway with reduced formation of fermentation byproducts

**DOI:** 10.1186/s12934-016-0612-6

**Published:** 2016-12-23

**Authors:** Jung-Hun Kim, Chonglong Wang, Hui-Jung Jang, Myeong-Seok Cha, Ju-Eon Park, Seon-Yeong Jo, Eui-Sung Choi, Seon-Won Kim

**Affiliations:** 1 Division of Applied Life Science (BK21 Plus), PMBBRC, Institute of Agricultural and Life Science, Gyeongsang National University, Jinju, 52828 South Korea; 2 Research Center for Industrial Chemical Biotechnology, KRICT, Ulsan, 44468 South Korea; 3 Life Science Research Institute, Daewoong Pharmaceutical Corporation, Yongin, South Korea; 4 Industrial Biotechnology Research Center, KRIBB, Daejeon, 305-806 South Korea

**Keywords:** Bioisoprene, Mevalonate pathway, Isoprene synthase, *Escherichia coli*, Carbon utilization

## Abstract

**Background:**

Isoprene, a volatile C5 hydrocarbon, is an important platform chemical used in the manufacturing of synthetic rubber for tires and various other applications, such as elastomers and adhesives.

**Results:**

In this study, *Escherichia coli* MG1655 harboring *Populus trichocarpa* isoprene synthase (Pt*ispS*) and the exogenous mevalonate (MVA) pathway produced 80 mg/L isoprene. Codon optimization and optimal expression of the *ispS* gene via adjustment of the RBS strength and inducer concentration increased isoprene production to 199 and 337 mg/L, respectively. To augment expression of MVA pathway genes, the MVA pathway was cloned on a high-copy plasmid (pBR322 origin) with a strong promoter (P_trc_), which resulted in an additional increase in isoprene production up to 956 mg/L. To reduce the formation of byproducts derived from acetyl-CoA (an initial substrate of the MVA pathway), nine relevant genes were deleted to generate the *E. coli* AceCo strain (*E. coli* MG1655 Δ*ackA*-*pta*, *poxB*, *ldhA*, *dld*, *adhE*, *pps*, and *atoDA*). The AceCo strain harboring the *ispS* gene and MVA pathway showed enhanced isoprene production of 1832 mg/L in flask culture with reduced accumulation of byproducts.

**Conclusions:**

We achieved a 23-fold increase in isoprene production by codon optimization of Pt*ispS*, augmentation of the MVA pathway, and deletion of genes involved in byproduct formation.

**Electronic supplementary material:**

The online version of this article (doi:10.1186/s12934-016-0612-6) contains supplementary material, which is available to authorized users.

## Background

Isoprene (2-methyl-1,3-butadiene) is an important feedstock for commercial production of synthetic rubber. Moreover, isoprene has higher energy content than other biofuels and is convertible to biofuel blend stocks, such as C10 gasoline, C15 diesel, and jet fuels [1]. Currently, 800,000 tons of isoprene monomer is produced annually from crude oil refineries, and over 95% of isoprene is used to produce cis-1,4-polyisoprene, a synthetic version of natural rubber [2]. As consumption of synthetic rubber has increased in the past decade, the demand for isoprene has also dramatically increased.

Currently, petroleum-based isoprene is the dominant source of isoprene available commercially. However, sustainable supply of isoprene is affected by fluctuations in the price of crude oil, high refining cost and energy consumption, and low recovery yield of pure isoprene from light gases produced by oil cracking (less than 1.7%) [3]. Moreover, the process used to prepare pure isoprene is thought to accelerate greenhouse gas emissions and global warming. As an alternative, biological isoprene (bioisoprene) production has been developing rapidly during the last decade.

**Fig. 1:**
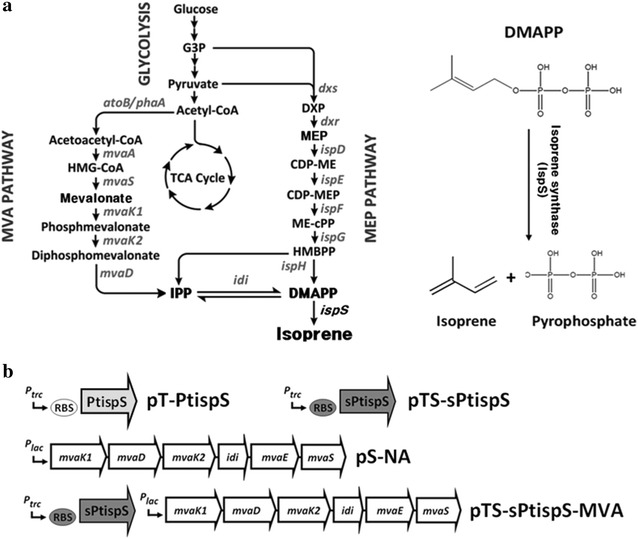
Isoprene synthesis pathway (**a**) and gene clusters used in this study (**b**). The *light* and *dark gray arrows* represent the wild-type isoprene synthase (PtispS) from *Populus trichocarpa* and its codon-optimized *ispS* (sPtispS) for expression in *Escherichia coli*, respectively. The *white* and *dark gray ovals* indicate the original RBS of the pTrc99A vector and the modified RBS with higher ribosomal affinity, respectively. The *white arrows* represent the genes of the MVA pathway (*mvaK1*, *mvaD*, and *mvaK2* from *Streptococcus pneumoniae*; *mvaE* and *mvaS* from *Enterococcus faecalis*; and *idi* from *Escherichia coli*)

Bioisoprene is synthesized by isoprene synthase from dimethylallyl diphosphate (DMAPP) [4], which is derived from the mevalonate (MVA) or methylerythritol phosphate (MEP) pathways (Fig. 1a) [5, 6]. Isoprene synthases have been isolated and characterized from various plant species [7–11], and allows microbial engineering for the production of isoprene [12]. Several reports have described isoprene production using *Cyanobacteria*, yeast, and *Bacillus* species engineered to overexpress plant isoprene synthases or the recombinant MVA or MEP pathway [13–15]. Although isoprene has been produced from these engineered microorganisms, the production titer is too low to meet industrial demand. *Bacillus* sp. are known to natively produce isoprene, and endogenous IspH from the MEP pathway can catalyze isoprene formation from HMBPP [16]. The *Bacillus* sp. N16-5 strain, which harbors an engineered IspH variant, can produce isoprene at concentrations of up to 352 μg/L/OD. *Saccharomyces cerevisiae* has been extensively engineered to produce isoprene, resulting in production of 37 mg/L isoprene. Successful isoprene production of 1.26 g/L from CO_2_ was also obtained following extensive engineering of the cyanobacterium *Synechococcus elongates* [17]. However, *Escherichia coli* is currently considered the most promising bacterial host for isoprene production. Zhao et al. [15] constructed an isoprene synthesis pathway in *Escherichia coli* based on the endogenous MEP pathway by overexpression of the native 1-deoxy-d-xylulose-5-phosphate (DXP) synthase gene (*dxs*) and the foreign DXP reductoisomerase gene (*dxr*) from *Bacillus subtilis* combined with introduction of the *ispS* gene from *Populus nigra*, which resulted in isoprene production of 314 mg/L. Isoprene has also been produced at 532 mg/L from recombinant *Escherichia coli* harboring the *ispS* gene from *Populus alba* and the exogenous MVA pathway from *Saccharomyces cerevisiae* [18]. In another report, the MVA pathway was improved by replacing the upper pathway from *Saccharomyces cerevisiae* with that of *Enterococcus faecalis*, which is more efficient [19], and introducing a single amino acid mutation in the *Enterococcus faecalis mvaS* gene to increase enzyme activity [20]. An *Escherichia coli* strain harboring the improved MVA pathway produced isoprene at up to 1.09 g/L in flask culture. An exogenous MVA pathway, composed of two different sources of MVA pathway from *Enterococcus faecalis* and *Streptococcus pneumoniae*, was also used for isoprene production from recombinant *Escherichia coli* overexpressing *Pueraria montana* (*Kudzu vine*) *ispS*, resulted in isoprene production of 320 mg/L [21]. Whited et al. achieved heterologous expression of *Populus alba ispS* and a bacterial/yeast hybrid MVA pathway containing additional *Methanosarcina mazei mvk*, together with overexpression of the endogenous *pgl* gene (encoding phosphogluconolactonase in the pentose phosphate pathway) [22]. The engineered *Escherichia coli* strain was able to produce 60 g/L isoprene in a bioreactor fed-batch culture after optimization of fermentation conditions.

In this study, we constructed the isoprene synthesis pathway with the *ispS* gene from *Populus trichocarpa* and the bacterial hybrid MVA pathway composed of two different genetic sources of *Streptococcus pneumoniae* and *Enterococcus faecalis* [19] (Fig. 1b). Expression of both *Populus trichocarpa ispS* and the hybrid exogenous MVA pathway in *Escherichia coli* were optimized and balanced to maximize isoprene production. Moreover, the genome of the host *Escherichia coli* strain was also engineered to reduce byproduct formation. The combination of pathway engineering of isoprene biosynthesis and genome manipulation resulted in a significant improvement in isoprene production.

## Results and discussion

### Comparison of isoprene production by isoprene synthases

**Fig. 2:**
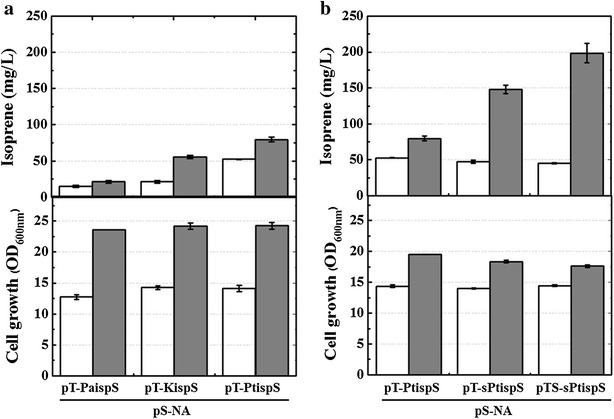
**a** Comparison of three different isoprene synthases (*Populus alba*, *Pueraria montana* [*Kudzu vine*], and *Populus trichocarpa*) for isoprene production from recombinant *Escherichia coli* strains harboring the MVA pathway. **b** Effects of codon optimization and the strong RBS for expression of *Populus trichocarpa* IspS on isoprene production and cell growth. Culture was carried out in TB medium containing 2.0% (w/v) glycerol for 24 h at 30 °C. *Open bars* and *closed bars* represent the values obtained at 12 and 24 h of culture, respectively

To determine the isoprene productivity of *ispS* genes from different plants, we compared three isoprene synthases from *Populus alba*, *Pueraria montana* (Kudzu vine) and *Populus trichocarpa*. The isoprene synthases of *Populus alba* and *Pueraria montana* have been used in most microbial engineering processes for isoprene production. However, the Km values of *Populus alba* and *Pueraria montana* isoprene synthases are relatively high (8.7 and 7.7 mM, respectively) [9, 21]. *Populus trichocarpa* isoprene synthase has not been used for microbial isoprene production, and its Km value is very low (0.32 mM) [11]. These three isoprene synthase genes were cloned into the pTrc99A vector, yielding pT-PaispS, pT-KispS, and pT-PtispS, respectively (Table 1). The three plasmids were introduced into *Escherichia coli* MG1655 with the MVA pathway plasmid of pS-NA to generate the recombinant strains MGpPapM, MGpKpM, and MGpPtpM, respectively (Table 1). The strain MGpPtpM harboring pT-PtispS and pS-NA produced 80 mg/L isoprene after 24 h of culture, which was 3.7- and 1.4-fold higher than those of the strains MGpPapM (pT-PaispS and pS-NA) and MGpKpM (pT-KispS and pS-NA), respectively, although there was no significant difference in cell growth among the three recombinant strains (Fig. 2a). The IspS from *Populus trichocarpa* was found to be superior to the other two isoprene synthases as a biocatalyst for isoprene production in *Escherichia coli*. Thus, the expression of *Populus trichocarpa* IspS combined with MVA pathway genes was optimized to enhance the production of isoprene.

**Table 1:** Primers, plasmids, and *Escherichia coli* strains used in this study

Names	Descriptions	References
Primers^a^		This study
PaispS-F	AGACGGTCTGCCAATTATGAACC	This study
PaispS-R	CTCTAGATTATCTCTCAAAGGGTAGAATAG	This study
PtispS-F	GCC **ATG** GCATGTTCTGTAAGCACAG	This study
PtispS-R	CTCTAGATTATCTCTCAAAGGGTAGAATAG	This study
KispS-F	TCTCTGGAAAATGACCTTAAGG	This study
KispS-R	CTCTAGATTAGCAGCCGGATCCCACGTAC	This study
sPtispS-F	GCC **ATG** GCTTGCTCTGTATCCAC	This study
sPtispS-R	CTCTAGATTAGCGTTCGAACGGCAGAATTG	This study
MVA-F	GTCTAGATACCTGACGCTTTTTATCGCAAC	This study
MVA-R	GTCTAGAGTTTCGATAAGAGCGAACGG	This study
Plasmids		
pTrc99A	P_trc_ expression vector, pBR322 origin, lacI^q^, Amp^r^	[23]
pTrc99S	pTrc99A containing strong RBS	This study
pSTV28	P_lac_ expression vector, pACYC184 origin, *lacZ*, Cm^r^	Takara
pT-PaispS	pTrc99A containing *ispS* from *P. alba*	This study
pT-PtispS	pTrc99A containing *ispS* from *P. trichocarpa*	This study
pT-KispS	pTrc99A containing *ispS* from *Kudzu vine*	This study
pT-sPtispS	pTrc99A containing codon optimized *ispS* from *P. trichocarpa*	This study
pTS-sPtispS	pTrc99S containing codon optimized *ispS* from *P. trichocarpa*	This study
pTS-sPt-MVA	pTS-sPtispS containing MVA pathway from plasmid pS-NA	This study
pS-NA	pSTV28 containing *mvaE* and *mvaS* from *E. faecalis*; *mvaK1*, *mvaK2*, and *mvaD* from *S. pneumoniae*; and *idi* from *E. Coli*	[19]
Strains		
MG1655	*E. coli* K-12; F^−^λ^−^, ilvG^−^, *rfb*-50, *rph*-1	ATCC 700926
AceCo	MG1655 Δ*ackA*-*pta, poxB, ldhA, dld, adhE, pps, atoDA*	Additional file 1: Table S1
DH5α	*E. coli* K-12; F^−^, φ80d*lacZ*ΔM15, Δ(*lacZYA*-*argF*)U169, *deoR*, *recA1*, *endA1*, *hsdR*17 (rK^−^ mK^+^), *phoA*, *supE*44, λ^−^, *thi*-1	ATCC 98040
MGpPapM	MG1655 harboring pT-PaispS and pS-NA	This study
MGpKpM	MG1655 harboring pT-KispS and pS-NA	This study
MGpPtpM	MG1655 harboring pT-PtispS and pS-NA	This study
MGpsPtpM	MG1655 harboring pTS-sPtispS and pS-NA	This study
MGpsPtM	MG1655 harboring pTS-sPtispS-MVA	This study
ApsPtM	AceCo harboring pTS-sPtispS-MVA	This study

^a^Start codons are presented in bold, and restriction sites are underlined

### Optimization of *Populus trichocarpa ispS* and MVA pathway gene expression

**Fig. 3:**
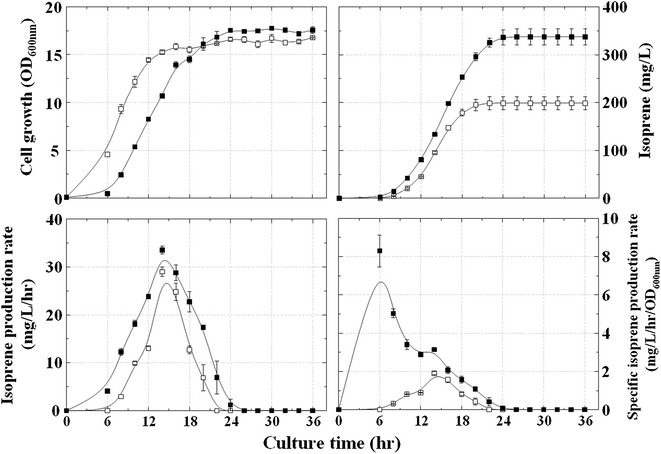
Effects of IPTG induction on isoprene production and cell growth of the MGpsPtpM strain (MG1655 harboring pTS-sPtispS and pS-NA). Culture was carried out in TB medium containing 2.0% (w/v) glycerol without IPTG (*open squares*) or with 0.1 mM IPTG (*closed squares*) for 36 h at 30 °C

To enhance the expression of the *Populus trichocarpa ispS* gene, the gene was codon-optimized and cloned into pTrc99A and pTrc99S (modified pTrc99A with a strong RBS), yielding pT-sPtispS and pTS-sPtispS, respectively (Table 1). The strain MGpsPtpM, which harbored pTS-sPtispS and pS-NA, produced 199 mg/L isoprene, which was increased by 2.5-fold from that of the strain MGpPtpM (pT-PtispS and pS-NA; Fig. 2b), suggesting that the enhanced expression of *Populus trichocarpa ispS* resulting from codon optimization and the use of a strong RBS significantly increased isoprene production. Thus, further overexpression of isoprene synthase combined with MVA pathway genes was stimulated by isopropyl β-d-1-thiogalactopyranoside (IPTG) induction. The strain MGpsPtpM (pTS-sPtispS and pS-NA) was induced with 0.1 mM IPTG, which resulted in an additional 1.7-fold increase in isoprene production to 327 mg/L (Fig. 3). These data indicated that overexpression of both IspS and the MVA pathway increased the synthesis of isoprene in the engineered *Escherichia coli*. However, a significant reduction in cell growth was observed with the addition of 0.1 mM IPTG. This growth inhibition was thought to be caused by shortage of DMAPP, which would occur in the strain MGpsPtpM owing to the increased overexpression of IspS from pTS-sPtispS rather than the MVA pathway from pS-NA in the presence of IPTG. Therefore, an increased supply of DMAPP through augmentation of the MVA pathway was required for both recovery of cell growth and achievement of high isoprene production.

**Fig. 4:**
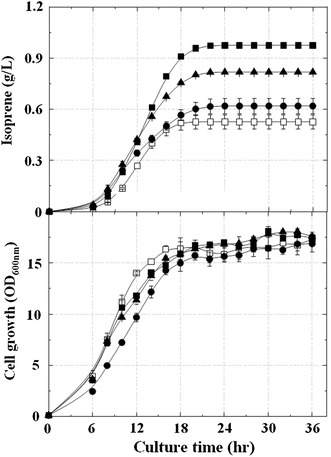
Effects of low IPTG concentrations on isoprene production and cell growth of the MGpsPtM strain (MG1655 harboring pTS-sPtispS-MVA). Culture was carried out in TB medium containing 2.0% (w/v) glycerol for 36 h at 30 °C. IPTG was initially added at concentrations of 0 mM (*open squares*), 0.01 mM (*closed squares*), 0.02 mM (*closed triangles*), and 0.03 mM IPTG (*closed circles*)

The MVA pathway operon in pS-NA, which exhibited a low copy number and a mild *lac* promoter, was ligated into the isoprene plasmid pTS-sPtispS with a high copy number and a strong *trc* promoter, yielding pTS-sPtispS-MVA (Fig. 1b; Table 1). The strain MGpsPtM harboring pTS-sPtispS-MVA was cultured and induced with 0.05 and 0.1 mM IPTG to observe the effects of overexpression of the MVA pathway on both isoprene production and cell growth (Additional file 1: Figure S3). Under the induction conditions using 0.05 and 0.1 mM IPTG, cell growth was inhibited, even when isoprene production was not increased. These results may be related to the cellular toxicity arising from intracellular accumulation of IPP and DMAPP owing to the overexpression of the MVA pathway from pTS-sPtispS-MVA [23]. Hence, the culture was carried out with the lower concentrations of IPTG (0.01–0.03 mM; Fig. 4). Although there were no significant difference in cell growth depending on the tested IPTG concentrations, isoprene production varied considerably with the concentration of IPTG, and the highest yield of 974 mg/L was obtained at 0.01 mM IPTG, which was 1.7-fold higher than that obtained in the absence of IPTG (the isoprene production rate and culture broth pH are presented in Additional file 1: Figure S4). Thus, the optimal IPTG concentration for culture induction was found to be 0.01 mM. These results suggested that the overall metabolic balance achieved by optimized overexpression of IspS and the MVA pathway was an important element for isoprene production.

### Analysis of byproducts in culture broth of MGpsPtM

Isoprene production was increased by 12-fold (80–974 mg/L) through optimized overexpression of the IspS and MVA pathway. However, the isoprene production titer was still low when considering the amount of carbon source used in the culture, and the culture pH was significantly reduced from 7.0 to 5.3. The formation of acetate, a representative acidic byproduct of *Escherichia coli*, was analyzed from culture broth of the strain MGpsPtM induced by 0.01 mM IPTG (Additional file 1: Figure S5). Acetate was steadily produced to a final amount of 7.4 g/L during the culture. These data suggested that a significant amount of acetyl-CoA was wasted in the production of acetate rather than isoprene. In order to stimulate production of isoprene in the recombinant *Escherichia coli* without wasting the carbon source, it may be necessary to prevent the formation of byproducts, such as acetate, by the production strain through genomic manipulation, permitting conservation of the acetyl-CoA pool for production of isoprene through the MVA pathway.

### Enhanced isoprene production through reduced byproduct formation in the AceCo strain

**Fig. 5:**
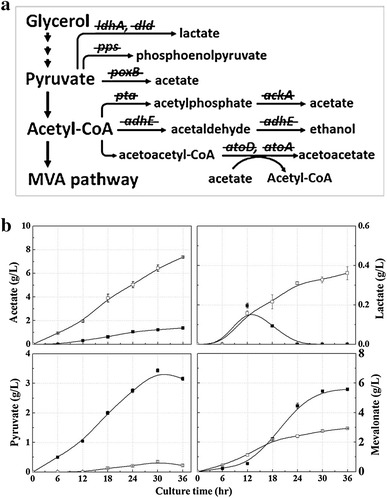
**a** Pathway of byproduct formation from acetyl-CoA and pyruvate in *Escherichia coli*. *Strikeouts* indicate deleted genes in the AceCo strain. The deleted genes and their corresponding enzymes are as follows: *ldhA* lactate dehydrogenase; *dld* d-lactate dehydrogenase; *pps* phosphoenolpyruvate synthetase; *poxB* pyruvate oxidase; *adhE* aldehyde-alcohol dehydrogenase; *pta* phosphate acetyltransferase; *ackA* acetate kinase; *atoD* acetoacetyl-CoA transferase; *atoA* acetoacetyl-CoA transferase. **b** Analysis of extracellular metabolites (acetate, lactate, pyruvate, and mevalonate) accumulated during the culture of strain MGpsPtM (wild-type MG1655 harboring pTS-sPtispS-MVA) shown in Fig. 4 (*open squares*) and the strain ApsPtM (the knockout mutant AceCo harboring the same plasmid) shown in Fig. 6 (*closed squares*) after induction with 0.01 mM IPTG

**Fig. 6:**
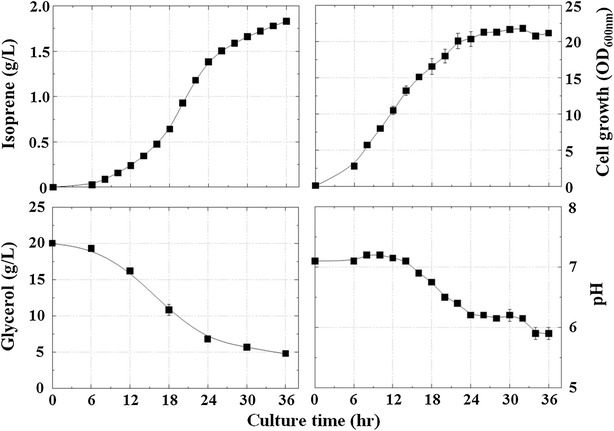
Effects of deletion of genes involved in byproduct formation on isoprene production. The strain ApsPtM (AceCo harboring pTS-sPtispS-MVA) was cultivated in TB medium containing 2.0% (w/v) glycerol and 0.01 mM IPTG at 30 °C

To reduce carbon source waste and increase carbon flow to isoprene production, we deleted nine genes involved in the formation of byproducts, including acetate, generating the knockout mutant strain AceCo (Table 1; Fig. 5a; Additional file 1: Table S1). The deleted genes (*ackA*-*pta*, *poxB*, *ldhA*, *dld*, *adhE*, *pps*, and *atoDA*)were selected to increase carbon flow to the MVA pathway by maximizing the level of acetyl-CoA through preventing formation of the byproducts derived from acetyl-CoA and its precursor, pyruvate. The *ackA*-*pta* and *poxB* genes were deleted to prevent formation of acetate. At the same time, deletions of *ldhA*/*dld* and *adhE* genes were carried out to prevent formation of lactate and ethanol, respectively. The genes *pps* and *atoDA* were deleted to prevent the gluconeogenic conversion of pyruvate to phosphoenolpyruvate and the dissipation of the MVA pathway intermediate acetoacetyl-CoA to acetoacetate, respectively. The strain ApsPtM (the AceCo mutant transformed with pTS-sPtispS-MVA) was cultivated to investigate isoprene production and byproduct formation (Figs. 5b, 6). The ApsPtM strain produced 1832 mg/L of isoprene, which was about twofold higher than the production of the MGpsPtM strain, although the ApsPtM strain consumed less glycerol (4.8 g/L of the final residual glycerol concentration) compared with the complete glycerol consumption of the MGpsPtM strain (Additional file 1: Figure S5). Acetate formation was significantly reduced to 1.4 g/L in strain ApsPtM compared with 7.4 g/L in the strain MGpsPtM. Lactate is a minor byproduct formed in small amounts of less than 0.4 g/L for both strains, although lactate formation by strain ApsPtM was lower than that by strain MGpsPtM. Interestingly, significant amounts of pyruvate (3.15 g/L) accumulated in the culture broth of the strain ApsPtM compared with that (0.22 g/L) of the strain MGpsPtM owing to secretion of the elevated pyruvate in the mutant by blockage of acetate and lactate formation. Accumulation of mevalonate (5.56 g/L) was higher in the culture broth of the mutant strain ApsPtM than that (2.93 g/L) of the wild-type strain MGpsPtM, which could reflect the elevation of acetyl-CoA in the mutant cells because one mevalonate molecule is produced from three acetyl-CoA molecules. There was no detectable accumulation of ethanol, PEP, or acetoacetate in the culture broth of strains MGpsPtM and ApsPtM (data not shown). These results indicated that the acetyl-CoA flux in the ApsPtM strain was directed to the MVA pathway by preventing generation of waste byproducts from pyruvate and acetyl-CoA.

## Conclusion

Taken together, our findings demonstrated that isoprene production could be increased by engineering of the isoprene synthesis pathway based on the MVA pathway and preventing byproduct formation through genome manipulation (Table 2). The combination of codon optimization of *Populus trichocarpa* isoprene synthase and the introduction of the heterologous MVA pathway resulted in a 4.2-fold increase in isoprene production. Construction of a single plasmid system by transfer of the MVA pathway from pS-NA to pTS-sPtispS, followed by optimal overexpression of these genes with 0.01 mM IPTG, resulted in an additional increase in isoprene production by 12.2-fold. The highest isoprene production of 1832 mg/L was achieved using the AceCo strain by reducing the formation of inhibitory byproducts, such as acetate. Although a considerable increase in isoprene production was attained in this study, mevalonate accumulation was also observed, suggesting that the bottom portion of the MVA pathway were less efficient than the top portion and that the pathway required further engineering to achieve higher efficiency. If the efficiency of the bottom portion in the MVA pathway is enhanced, mevalonate may not accumulate, and acetyl-CoA may flow more easily into the MVA pathway, resulting in further reductions in the accumulation of pyruvate in the AceCo strain by its favorable conversion to acetyl-CoA. Therefore, future studies are needed to engineer the bottom portion of the MVA pathway in order to increase isoprene production further. This may be achieved by identification of the rate limiting step in the bottom portion and replacement of inefficient enzymes with more efficient enzymes or by augmenting the overexpression of the enzyme.

**Table 2:** Stepwise increases in isoprene production from the engineered *Escherichia coli* strains

Strains	IPTG induction	Isoprene production (mg/L)	Fold increase
MGpPtpM	None	80	1.0
MGpsPtpM	None	199	2.5
MGpsPtpM	0.1 mM IPTG	337	4.2
MGpsPtM	None	526	6.6
MGpsPtM	0.01 mM IPTG	974	12.2
ApsPtM	0.01 mM IPTG	1832	23.0

## Methods

### Bacterial strains and culture conditions

The bacterial strains used in this study are listed in Table 1. *Escherichia coli* DH5α was used for gene cloning, and *Escherichia coli* MG1655 was used for isoprene production (Table 1). Culture of isoprene production was carried out in a 300-mL baffled flask containing 50 mL TB medium (24 g/L yeast extract, 12 g/L tryptone, 9.2 g/L K_2_HPO_4_, and 2.2 g/L KH_2_PO_4_) with 2.0% (w/v) glycerol as the main carbon source. IPTG was added at concentrations of 0.01–0.1 mM. Ampicillin (100 μg/mL) and chloramphenicol (50 μg/mL) were added to the culture as required. Cell growth was determined by measuring the optical density at 600 nm (OD_600_), and pH was measured using a compact pH meter (B-212; HORIBA, Japan).

### Plasmid and strain construction

The plasmids and polymerase chain reaction (PCR) primers used in this study are listed in Table 1. All basic molecular techniques, including genomic or plasmid DNA preparations, restriction enzyme digestions, and transformations, were carried out as described in literature. PCR was performed using *Phusion* DNA polymerase (Finnzymes Co., Finland) with a standard protocol. pTrc99A (RBS and gap sequences: AGGAAACAGA), pTrc99S (a modified pTrc99A version with a strong RBS and gap sequences: AGGAGGTAATAAA), and pSTV28 plasmids were used for gene expression (Table 1). Translation initiation rates (TIRs) of *ispS* were 217 and 39,327 for pTrc99A and pTrc99S vectors, respectively. The TIR was calculated with the RBS calculator program (https://salislab.net/; Salis Lab, Penn State University, PA, USA). The pS-NA plasmid containing the MVA pathway operon was used as described previously [19]. Three different *ispS* genes lacking the transit peptide sequence were amplified using PCR from plasmids harboring *ispS* genes of *Populus alba*, *Populus trichocarpa*, and *Pueraria montana*, which were kindly provided by Prof. Claudia E. Vickers (University of Queensland, Australia). The PCR products were cloned into the *Nco*I (followed by Klenow treatment for cloning of *ispS* genes from *Populus alba* and *Pueraria montana*) and *Xba*I sites of pTrc99A, yielding the isoprene plasmids pT-PaispS, pT-PtispS, and pT-KispS (Table 1). The *Populus trichocarpa ispS* gene (GenBank accession number: EU693027) was also synthesized by Genscript Inc. (NJ, USA) according to the codon-optimization function of the company’s in-house software for enhanced expression in *Escherichia coli.* The synthetic *ispS* gene was cloned into the *Nco*I and *Xba*I sites of pTrc99A and pTrc99S, yielding pT-sPtispS and pTS-sPtispS, respectively. The MVA pathway operon from pS-NA was amplified and cloned into the *Xba*I site of pTS-sPtispS, which resulted in pTS-sPtispS-MVA.

The mutant strain, called AceCo, was derived from *Escherichia coli* MG1655 by deletions of nine genes related to the formation of inhibitory byproducts, such as alcohol and acids (Table 1). The AceCo strain (*Escherichia coli* MG1655 Δ*ackA*-*pta*, *poxB*, *ldhA*, *dld*, *adhE*, *pps*, and *atoDA*) was generated by combination of individual gene knockouts using P1 transduction with phage P1 (ATCC 25404-B1) [24]. The knockout strains and primers used for the construction of the AceCo strain are listed in Additional file 1: Table S1. The seven basal knockout strains (IS1–7) were generated using a gene deletion kit based on Red/ET recombination (Quick and Easy *E. coli* Gene Deletion Kit; Gene Bridges, Germany) with standard protocols.

### Analysis of isoprene, byproducts, and mevalonate

To measure isoprene production, isoprene produced from recombinant *Escherichia coli* was collected by overlaying a dodecane (C_12_H_26_) layer. Dodecane has properties of low toxicity to *Escherichia coli* [25], low volatility, and high hydrophobicity (log P_O/W_, 6.6) and is suitable for recovery of hydrophobic isoprene. The culture broth, 0.7 mL was sampled every 2 h and incubated with an equal volume of dodecane in 1.5 mL Eppendorf tube at 30 °C with shaking at 150 rpm for 10 min. The dodecane phase was separated from the culture broth by centrifugation at 10,000 rpm for 10 s. The separated dodecane phase containing isoprene was analyzed using an Agilent Technologies 7890A Gas Chromatograph equipped with a flame ionization detector (FID). Briefly, 5 μL of each sample was separated on a 19091N-133 HP-INNOWAX column (length, 30 m; internal diameter, 0.25 mm; film thickness, 250 μm). The oven temperature was initially held at 50 °C for 2 min and then gradually increased to 250 °C at a rate of 30 °C/min. Nitrogen was used as the carrier gas with an inlet pressure of 15.345 psi. The detector temperature was maintained at 280 °C. All the works were carried out in speedy to minimize volatile loss of isoprene. The retention time of the isoprene standard was 1.38 min (Additional file 1: Figure S1). The standard curve of isoprene was constructed using isoprene (Sigma, USA) dissolved in dodecane for quantification of isoprene production (Additional file 1: Figure S2). A sealed glass bottle was used for dilution of chilled standard isoprene to make the standard curve. The dilution work was done very quickly and the glass bottle was kept sealed except the quick addition of isoprene standards.

Byproducts, including acetate, lactate, pyruvate, and ethanol, in culture broth were analyzed with high-performance liquid chromatography (HPLC; LC-20A; Shimadzu, Kyoto, Japan) at a detection wavelength of 210 nm with an ion exchange HPLC column (Aminex; HPX-87H, 7.8 × 300 mm; Bio-Rad, USA). The mobile phase of 5 mM H_2_SO_4_ was used at a flow rate of 0.6 mL/min, and the column temperature of 40 °C was applied. The residual glycerol was analyzed by HPLC equipped with an RID detector (RID-10A; Shimadzu) and hydrophobic interaction HPLC column (100-5NH_2_, 250 × 4.6 mm; Kromasil, Sweden). The mobile phase of 75% acetonitrile was used at a flow rate of 1.5 mL/min, and a column temperature of 40 °C was applied for the analysis. Acetate, lactate, pyruvate, and ethanol were purchased from Sigma, dissolved in distilled water, and used as standard compounds.

The mevalonate concentration was determined by gas chromatography (GC) analysis. The culture filtrate was adjusted to pH 2.0 with 3 M HCl, incubated at 45 °C for 1 h, saturated with anhydrous Na_2_SO_4_, and extracted with ethylacetate. The resulting samples were analyzed for quantification of mevalonate using GC. The analytical temperature of the GC was controlled at an initial temperature of 180 °C for 1 min, then ramped to 200 °C gradually at 2.5 °C/min and held for 2 min. The detector temperature was maintained at 260 °C. The mevalonate standard compound was prepared from mevalonolactone (Sigma-Aldrich) as described in a previous report [26].

## Additional file

**Additional file 1: Table S1.** Primers and strains used for the construction of *E. coli* AceCo strain. **Figure S1.** Chromatogram of isoprene standard dissolved in dodecane. **Figure S2.** Standard curve of isoprene. **Figure S3.** Effect of IPTG induction on isoprene production and cell growth of MGpsPtM strain (MG1655 harboring pTS-sPtispS-MVA). **Figure S4.** Effect of low IPTG concentrations on isoprene production rate and culture broth pH in the culture of MGpsPtM strain (MG1655 harboring pTS-sPtispS-MVA). **Figure S5.** Acetate formation and glycerol consumption of the 0.01 mM IPTG induced culture of MGpsPtM in Fig. 4.

## Abbreviations

DMAPP: dimethylallyl diphosphate
MVA: mevalonate
MEP: methylerythritol 4-phosphate
DXP: 1-deoxy-d-xylulose-5-phosphate
IPP: isopentenyl diphosphate
TIR: translation initiation rate
