# Supplementary material for: Isoprene production by Escherichia coli through the exogenous mevalonate pathway with reduced formation of fermentation byproducts
Source: Microb Cell Fact. 2016 Dec 23;15:214. doi: 10.1186/s12934-016-0612-6 (PMC5180398; doi:10.1186/s12934-016-0612-6)
Supplement: Supplementary file 1 — Additional file 1: Table S1. Primers and strains used for the construction of E. coli AceCo strain. Figure S1. Chromatogram of isoprene standard dissolved in dodecane. Figure S2. Standard curve of isoprene. Figure S3. Effect of IPTG induction on isoprene production and cell growth of MGpsPtM strain (MG1655 harboring pTS-sPtispS-MVA). Figure S4. Effect of low IPTG concentrations on isoprene production rate and culture broth pH in the culture of MGpsPtM strain (MG1655 harboring pTS-sPtispS-MVA). Figure S5. Acetate formation and glycerol consumption of the 0.01 mM IPTG induced culture of MGpsPtM in Fig. 4. [file 12934_2016_612_MOESM1_ESM.doc]

**Additional file 1**

**Table S1.** Primers and strains used for construction of *E. coli* AceCo strain

| **Names** | **Descriptions** | **References** |
| --- | --- | --- |
| **Primers^*^** |  |  |
| ΔackA-pta-F | TGGCTCCCTGACGTTTTTTTAGCCACGTATCAATTATAGGTACTTCCA  TG**AATTAACCCTCACTAAAGGGCG** | This study |
| ΔackA-pta-R | GCAGCGCAAAGCTGCGGATGATGACGAGATTACTGCTGCTGTGCAGAC  TG**TAATACGACTCACTATAGGGCTC** | This study |
| ackA-ptaCF-F | **TGTCATCATGCGCTACGCTC** | This study |
| ackA-ptaCF-R | **CAGTTAAGCAAGATAATCAG** | This study |
| ΔpoxB-F | GATGAACTAAACTTGTTACCGTTATCACATTCAGGAGATGGAGAACCA  TG**AATTAACCCTCACTAAAGGGCG** | This study |
| ΔpoxB-R | CCTTATTATGACGGGAAATGCCACCCTTTTTACCTTAGCCAGTTTGTT  TT**TAATACGACTCACTATAGGGCTC** | This study |
| poxBCF-F | **TTACGTACTGGCCTGCTCCTGC** | This study |
| poxBCF-R | **GTCGGGTAACGGTATCACTGCG** | This study |
| ΔldhA-F | ATTTTTAGTAGCTTAAATGTGATTCAACATCACTGGAGAAAGTCTTAT  GA**AATTAACCCTCACTAAAGGGCG** | This study |
| ΔldhA-R | CTCCCCTGGAATGCAGGGGAGCGGCAAGATTAAACCAGTTCGTTCGGG  CA**TAATACGACTCACTATAGGGCTC** | This study |
| ldhACF-F | **TCATCAGCAGCGTCAACGGC** | This study |
| ldhACF-R | **CGCTGGTCACGGGCTTACCG** | This study |
| ΔadhE-F | CGAGCAGATGATTTACTAAAAAAGTTTAACATTATCAGGAGAGCATTA  TG**AATTAACCCTCACTAAAGGGCG** | This study |
| ΔadhE-R | CCGTTTATGTTGCCAGACAGCGCTACTGATTAAGCGGATTTTTTCGCT  TT**TAATACGACTCACTATAGGGCTC** | This study |
| adhECF-F | **CCGCACTGACTATACTCTCG** | This study |
| adhECF-R | **TGATCGGCATTGCCCAGAAG** | This study |
| ΔatoDA-F | CTATTGCCTGACTGTACCCACAACGGTGTATGCAAGAGGGATAAAAAA  TG**AATTAACCCTCACTAAAGGGCG** | This study |
| ΔatoDA-R | ACGCGTCATAAAACGCGATATGCGACCAATCATAAATCACCCCGTTGC  GTT**TAATACGACTCACTATAGGGCTC** | This study |
| atoDACF-F | **TGGCGAGGTAAAAACAGCCCC** | This study |
| atoDACF-R | **AAGCGCGATCACGAATGTTAGC** | This study |
| Δdld-F | CGCTATTCTAGTTTGTGATATTTTTTCGCCACCACAAGGAGTGGAAAA  TG**AATTAACCCTCACTAAAGGGCG** | This study |
| Δdld-R | GGATGGCGATACTCTGCCATCCGTAATTTTTACTCCACTTCCTGCCAG  TT**TAATACGACTCACTATAGGGCTC** | This study |
| dldCF-F | **CAGACTCACCGCGATTCCTACTG** | This study |
| dldCF-R | **CGGTAAAGTGATGCCTGTGCC** | This study |
| Δpps-F | AGAAATGTGTTTCTCAAACCGTTCATTTATCACAAAAGGATTGTTCGA  TG**AATTAACCCTCACTAAAGGGCG** | This study |
| Δpps-R | CGGCGACTAAACGCCGCCGGGGATTTATTTTATTTCTTCAGTTCAGCC  AGT**TAATACGACTCACTATAGGGCTC** | This study |
| ppsCF-F | **GCAGATTTGCGCAACGCTGG** | This study |
| ppsCF-R | **CTGCCGTATGGATGAGGCTGG** | This study |
| **Strains** |  |  |
| IS1 | *E. coli* MG1655 Δ*ackA-pta* | This study |
| IS2 | *E. coli* MG1655 Δ*poxB* | This study |
| IS3 | *E. coli* MG1655 Δ*ldhA* | This study |
| IS4 | *E. coli* MG1655 Δ*dld* | This study |
| IS5 | *E. coli* MG1655 Δ*adhE* | This study |
| IS6 | *E. coli* MG1655 Δ*pps* | This study |
| IS7 | *E. coli* MG1655 Δ*atoDA* | This study |
| IS8 | *E. coli* MG1655 Δ*ackA-pta, poxB* **(IS1 + IS2)** | This study |
| IS9 | *E. coli* MG1655Δ*ackA-pta, poxB, ldhA* **(IS8 + IS3)** | This study |
| IS10 | *E. coli* MG1655Δ*ackA-pta, poxB, ldhA, dld* **(IS9 + IS4)** | This study |
| IS11 | *E. coli* MG1655Δ*ackA-pta, poxB, ldhA, dld, adhE* **(IS10 + IS5)** | This study |
| IS12 | *E. coli* MG1655Δ*ackA-pta, poxB, ldhA, dld, adhE, pps* **(IS11 + IS6)** | This study |
| AceCo | *E. coli* MG1655Δ*ackA-pta, poxB, ldhA, dld, adhE, pps, atoDA* **(IS12 + IS7)** | This study |

* Bold letters of primer sequences represent binding sequence to template DNA.


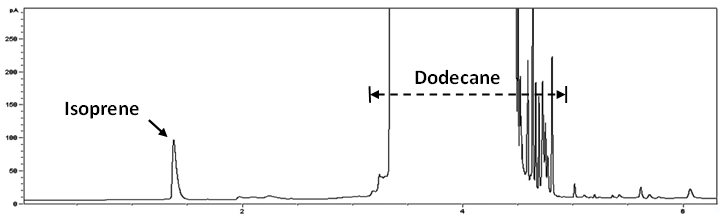


**Figure S1.** Chromatogram of isoprene standard dissolved in dodecane. The retention time of isoprene is 1.38 min.


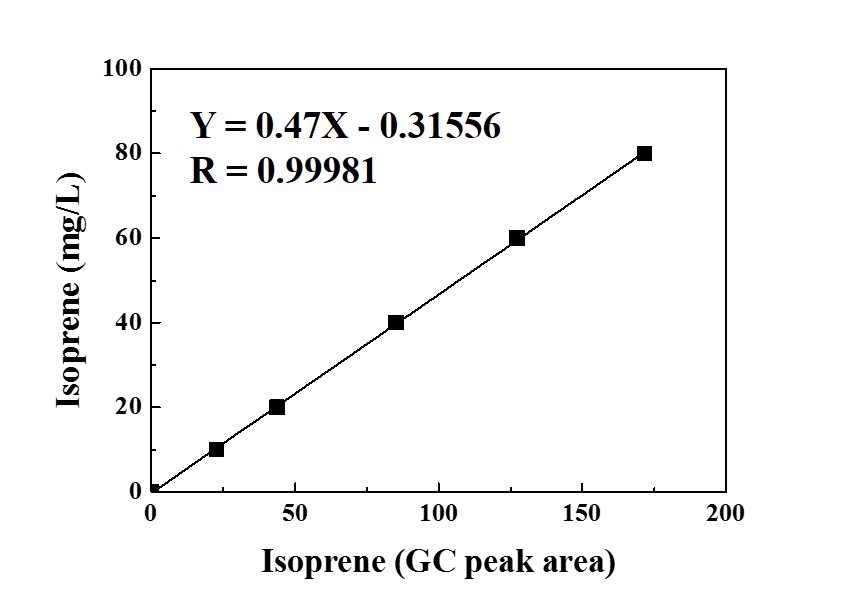


**Figure S2.** Standard curve of isoprene. X, Y and R represent GC peak area, isoprene concentration and regression coefficient, respectively.


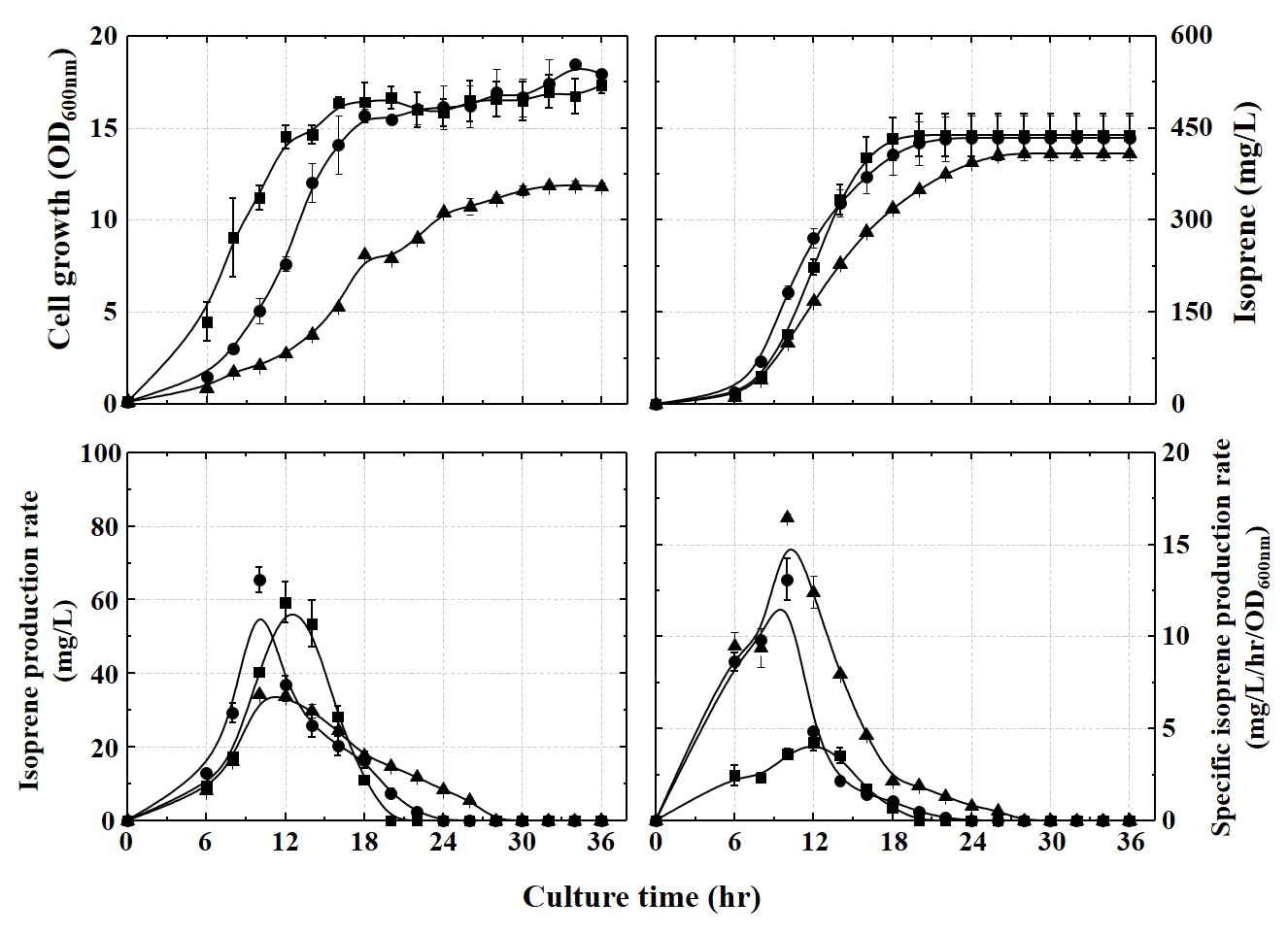


**Figure S3.** Effect of IPTG induction on isoprene production and cell growth of MGpsPtpM strain (MG1655 harboring pTS-sPtispS-MVA). Culture was carried out in TB medium containing 2.0 % (w/v) glycerol with 0 mM (square), 0.05 mM (circle) and 0.1 mM (triangle) IPTG inductions for 36 hours at 30 °C.

**
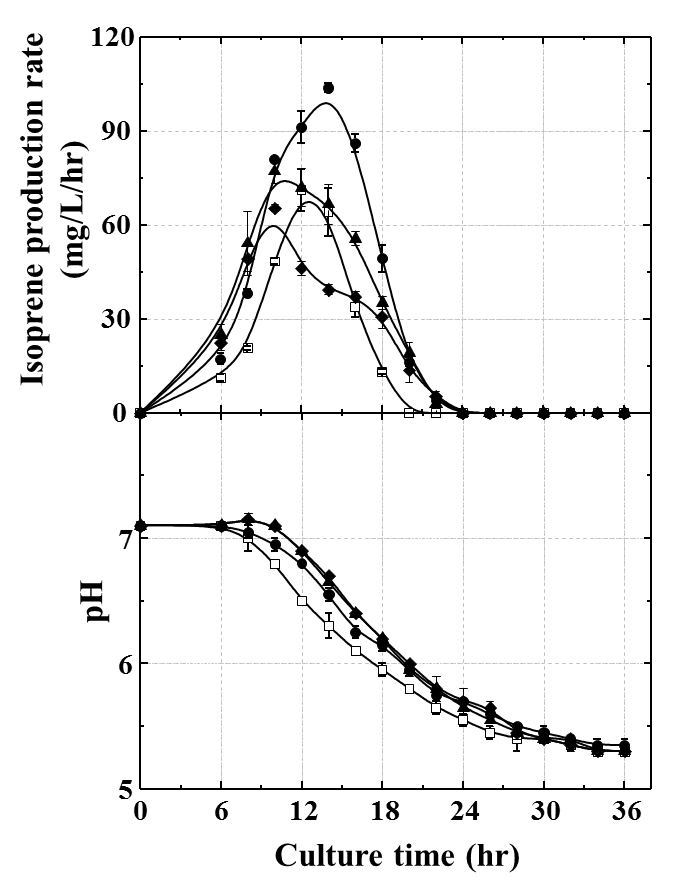
**

**Figure S4.** Effect of low IPTG concentrations on isoprene production rate and culture broth pH in the culture of MGpsPtM strain (MG1655 harboring pTS-sPtispS-MVA). Culture was carried out in TB medium containing 2.0 % (w/v) glycerol with 0 mM (open square), 0.01 mM (closed square), 0.02 mM (closed triangle) and 0.03 mM (closed circle) IPTG inductions for 36 hours at 30 °C.


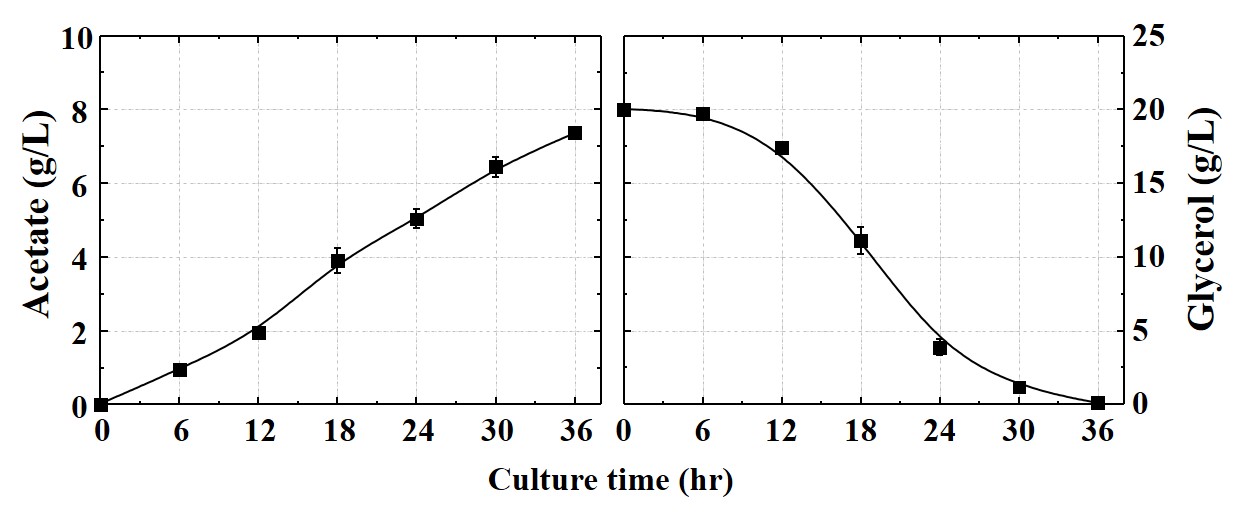


**Figure S5.** Acetate formation and glycerol consumption of the 0.01 mM IPTG induced culture of MGpsPtM in Figure 4.
